# Drift correction for single-molecule imaging by molecular constraint field, a distance minimum metric

**DOI:** 10.1186/s13628-014-0015-1

**Published:** 2015-01-13

**Authors:** Renmin Han, Liansan Wang, Fan Xu, Yongdeng Zhang, Mingshu Zhang, Zhiyong Liu, Fei Ren, Fa Zhang

**Affiliations:** Key Lab of Intelligent Information Processing and Advanced Computing Research Lab, Institute of Computing Technology, Chinese Academy of Sciences, Beijing, 100190 China; University of Chinese Academy of Sciences, Beijing, China; Beijing Institute of Technology, Beijing, China; Institute of Biophysics, Chinese Academy of Sciences, Beijing, 100101 China; State Key Lab for Computer Architecture, Institute of Computing Technology, Chinese Academy of Sciences, Beijing, 100190 China

**Keywords:** Image reconstruction techniques, Resolution, Fluorescence microscopy, Superresolution

## Abstract

**Background:**

The recent developments of far-field optical microscopy (single molecule imaging techniques) have overcome the diffraction barrier of light and improve image resolution by a factor of ten compared with conventional light microscopy. These techniques utilize the stochastic switching of probe molecules to overcome the diffraction limit and determine the precise localizations of molecules, which often requires a long image acquisition time. However, long acquisition times increase the risk of sample drift. In the case of high resolution microscopy, sample drift would decrease the image resolution.

**Results:**

In this paper, we propose a novel metric based on the distance between molecules to solve the drift correction. The proposed metric directly uses the position information of molecules to estimate the frame drift. We also designed an algorithm to implement the metric for the general application of drift correction. There are two advantages of our method: First, because our method does not require space binning of positions of molecules but directly operates on the positions, it is more natural for single molecule imaging techniques. Second, our method can estimate drift with a small number of positions in each temporal bin, which may extend its potential application.

**Conclusions:**

The effectiveness of our method has been demonstrated by both simulated data and experiments on single molecular images.

## Background

Far-field optical microscopy has played an important role in the biological sciences. However, the imaging capacity of a conventional light microscope is fundamentally limited by the wavelength of the light. Recent developments in optical microscopy, such as fluorescence photoactivation localization microscopy(FPALM/PALM) [1] and stochastic optical reconstruction microscopy(STORM) [2,3], have overcome the diffraction limit [4] and achieved resolution values down to 20 nm, a factor of ten lower than the resolution of conventional light microscopy. These techniques exploit the stochastic photoswitching of fluorescent probe molecules and carefully choose imaging parameters to guarantee that no molecules closer together than the point spread function (PSF) width would emit signal at the same time. Because these techniques should readout a sequence of camera frames in a diffraction limited situation for the reconstruction of a super-resolution image, it is common that the image acquisition will take a long time. Typically, 1,000-100,000 individual camera frames [1,3,5,6] are needed, with exposure times of 1-100 ms per frame; this translates to several minutes to hours in total.

Long acquisition times increase the risk of sample drift during imaging [7]. Sample drift on the nanometer scale is hard to avoid as it may be caused by a variety of reasons, such as mechanical instability of instrument and vibration. Without correction, the lateral drift will smear the image and degenerate the resolution. The drift correction problem is a special case of image registration that has been discussed for decades in medical image processing and leads into a variety of solutions. Recently, several methods have been proposed to solve the drift correction problem in the field of single molecule imaging. Fiducial markers, typically gold nanoparticles or fluorescent beads, can be incorporated into the sample. Because the brightness of fiducial markers has a high contrast to that of probe molecules [1,3] and is stable during measurement, the positions of markers can be easily tracked and their trajectory can be used to correct the drift, which makes the method quite reliable. However, the fiducial marker-based method needs beads introduced into the sample and limits our observation area. Fiducial markers also may interfere with the sample and degenerate the localization precision of the nearby probe molecules [8]. To overcome the limitation of this marker-based method, cross-correlation is an alternative option. Cross-correlation utilizes the biological structure itself, which is stationary during the measurement, to estimate the drift. Though cross-correlation avoids the problems of introducing fiducial markers, there are also several shortcomings. Generally, the positions of molecules should be spatially binned as a 2D histogram to carry out the cross-correlation analysis [9,10]. The behavior of cross-correlation is close relative to the localized fluorescent position density of the 2D histogram. There are two decisive factors: the parameters of spatial binning and temporal binning. First, we should carefully choose the spatial binning parameter. If the spatial binning is too coarse, i.e., if the pixel width of the 2D histogram is too wide, smaller features of the molecular distribution will be lost. If the density of molecules in a pixel is too low, the histogram will not represent the global distribution of molecules, which may lead to the failure of the cross-correlation analysis. Second, the choice of the temporal binning parameter also determines the number of localized positions in a temporal bin. Typically, the number of positions in a temporal bin should be sufficient to give a meaningful description of molecule distribution. However, for applications where there are a small number of localized florescent events in each frame, the temporal bins will be too large to ignore the drift within one interval, which leads to an inaccurate estimation of the overall drift.

In this paper, we propose a new metric based on the distance between molecules to solve the drift correction. The proposed metric directly uses the position information of molecules to estimate the frame drift. Thus, the introduction of fiducial markers and the problems of binning the frames into 2D histogram are avoided. Since the metric directly operates on the positions, it is more natural and conceptually straight-forward for the single molecule imaging techniques. Though the choice of parameters of temporal binning is still a problem in our metric, by directly using the distance metric, just a small amount of information on the stable molecules can uncover the relationship between different frames. Therefore, the proposed method could estimate the drift with a small number of positions in each temporal bin, which may extend the potential application scenarios of our metric. We also designed an incremental algorithm to implement the metric for the general application of drift correction. Different experiments based on simulated data and real data have been carried out and the results demonstrate the effectiveness of our method.

## Methods

The stochastic switching of probe molecules is the fundamental mechanism of single molecule imaging. The switching on and switching off phenomenon of a single molecule obeys a Poisson process in the temporal domain [10]. Because the switching events of probes are asynchronous, the observation (frame) at different time points becomes a random sampling of the molecule distribution in the structure. In addition, considering the difference between the readout rate of microscopy and switching rate of probe, a florescent event observed in a frame may change or not be recorded in the next frame. Though the molecule position can be determined by various methods [11], the essence of these localization methods is fitting the distribution of the measured photon spot to that of the ideal point spread function (PSF) [12], which will unavoidably introduce localization error. Therefore, it is difficult to trace an identical molecule in different frames and we will not make any assumption about the traceability of probe molecules in our model.

The super-resolution image is reconstructed from the molecule positions. The localization precision of probe molecules has a great effect on the reconstruction quality. There are three kinds of uncertainties in the localization of probe molecules. The first uncertainty comes from the localization technique itself. The fitting methods are based on a probability model and certain assumption about the ideal PSF, but the fundamental assumption may not be completely consistent with the emitter properties in each application. In addition, the observed photon distribution may be affected by background noise or mutual interference of molecules, all of which affect the localization accuracy. The second uncertainty comes from the thermodynamic instability of probe molecules. The long acquisition of single molecule imaging gives a molecule enough chance to undergo random motion. The effect of random motion is intrinsic and inevitable in the period of measurement. The third uncertainty is the sample drift. These drifts will degenerate a reconstruction image to a great degree. Fortunately, they can be compensated by the drift correction method.

### Imaging model

The super-resolution image is reconstructed from all the molecule positions in the frames. Here, we introduce the concept of ‘position set’. A position set (denoted by Φ) is the set of all the localized positions measured in a time interval. If the experimental environment is ideal, Φ will be a random sampling of the signals that depict the biological structure. However, localization error, random motion of the probe molecule and sample drift complicate the situation. Thus, we define the measured molecule position $\vec {p}$ by

(1) $$\begin{array}{@{}rcl@{}} \vec{p}=\vec{p}_{\textit{ora}}+\vec{\phi}_{\textit{fit}}+\vec{\phi}_{\textit{ran}}+D \end{array} $$

where $\vec {p}_{\textit {ora}}$ is the corresponding position of probe molecule measured in the ideal condition (a position of oracle), $\vec {\phi }_{\textit {fit}}$ is the calculation error of photon distribution fitting, $\vec {\phi }_{\textit {ran}}$ is the uncertainty of random motion and *D* is the sample drift. In general, the calculation error obeys a Gaussian distribution, without considering the model bias. The probable positions of a probe molecule in random motion are also assumed to follow a Gaussian distribution, according to the most recent study and experiment by Nieuwenhuizen [13]. For convenience of discussion, we ignore their difference in mechanism and define them as a combination $\vec {\phi }$, i.e. $\vec {\phi }=\vec {\phi }_{\textit {fit}}+\vec {\phi }_{\textit {ran}}$ ($\vec {\phi }$ also obeys a Gaussian distribution). That is to say, if we already know the ideal position $\vec {p}_{\textit {ora}}$ of a florescent event and the drift *D*, the probability that a measured position *p* corresponds to the ideal position is:

(2) $$\begin{array}{@{}rcl@{}} Prob(\vec{p}) = \frac{1}{2\pi\sigma^{2}}exp\left(-\frac{\|\vec{p}-\vec{p}_{\textit{ora}}-D\|^{2}}{2\sigma^{2}}\right) \end{array} $$

where *σ* is the standard deviation of $\vec {\phi }$.

For a position set $\varPhi =\{\vec {p}_{j}|j=1,2,\ldots M\}$, the signal distribution of fluorescent probes can be described by the following density function:

(3) $$\begin{array}{@{}rcl@{}} \psi(\vec{x}) = \sum\limits_{i=1}^{M}{\delta\left(\vec{x}-\vec{p}_{j}\right)} \end{array} $$

where *δ*(·) is the Dirac delta function. The Dirac delta function can depict the signal distribution exactly, but there comes a problem when processing molecule position sets in which the sample drift has not been corrected. The Dirac delta function can not be used to judge the similarity between two position sets, because it does not formulate the uncertainty in the measurement. Here, we propose a novel metric to solve the problem. Our metric is based on the distance between molecules and can directly measure the similarity of signals by molecule positions.

**Figure 1:**
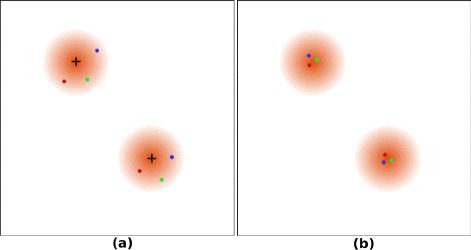
Illustration of the measured position set and drift correction in a structure composed of two emitters. **Illustration of the measured position set and drift correction in a structure composed of two emitters.** **(a)** Measured positions of a probe. The black cross denotes the ideal position of the probe. The blue, red and green points denote three measured positions of the probe in different times. **(b)** The corrected positions of the observed probe positions. The blue, red and green points denote the corrected positions of the corresponding observations in (a).

Let us consider the most simplified situation: a structure consists of two points. Figure 1 illustrates such a structure and on each point there is a florescent probe. There are three measured position sets obtained by observation and marked on Figure 1(a) by blue, red, and green points. All of the position sets have a measurable drift. The black cross denotes the ideal position of probe. We find that the observation data has deviated from the ideal position set a lot. Obviously, these three position sets are similar to each other, but the simple overlapping of the three position sets cannot depict the signal distribution successfully. In addition, such a data set cannot be corrected by the cross-correlation of a 2D histogram, because there are not enough positions. The measured signal is a random sampling of the true structure. Because the structure is only composed of two points, the drift can be corrected by distance (Euclidean distance) comparison of the corresponding position in each part. Figure 1(b) illustrates the relative position of these three position sets corrected by distance comparison.

As discussed above, the distance comparison is able to correct the drift of a structure composed of two points, but it does not work in more complex situation. However, we can extend the concept of distance comparison. First of all, we should make two basic rules clear in single molecule imaging techniques:

1. For different position sets that depict the identical biological structure, the distribution of their superset should be as sharp as possible.
2. For a given structure (distribution) that is depicted by molecule positions, the more positions there are, the better the signal strength output will be.

These rules are coincident with the criteria introduced in the recent resolution estimation work [13] and are also the guide line for drift correction. The distribution of molecule positions should obey the signal distribution of the underlying biological structure. Because of the measurement uncertainty, the molecule positions belong to different position sets, which represents the identical structure, but with slight difference. Here, we propose a new criterion, called the Point value of Molecular Constraint Field(PMCF), to indicate the sparseness of molecule positions around a spot. For a region centered on position *p*, the function is given by:

(4) $$\begin{array}{@{}rcl@{}} E (\vec{p})= \sum\limits_{\textit{region}}\text{FUN}\left(\|\vec{p}-\vec{p}_{j}\|\right),j=1,2\ldots M \end{array} $$

Where ∥·∥ is the *l*2−norm and $\vec {p}_{j}$ represents the point located in the region. The certain form of the kernel function FUN(·) has not been given out here. FUN(·) should be a function symmetric about the *y*-axis and depends on the real application. The summation of kernel function maps the distance between two points into a value describing the sparseness. According to the discussion of uncertainty of the image model, Gaussian function is chosen as the kernel function. Therefore, the generalized PMCF of position *p* is defined as follows:

(5) $$\begin{array}{@{}rcl@{}} PMCF(\vec{p}) = \sum\limits_{\textit{region}}\frac{1}{2\pi\sigma^{2}}exp\left(-\frac{\|\vec{p}-\vec{p}_{j}\|^{2}}{2\sigma^{2}}\right) \end{array} $$

Where $\vec {p}_{j}$ represents the probe molecules in region and *σ* restricts the effective width of kernel function. It is not hard to extend the idea from a molecule position to a position set. Assuming there are two position sets Φ_*A*_ and Φ_*B*_, the Molecular Constraint Field (MCF) of the given two position sets is defined as:

(6) $$\begin{array}{@{}rcl@{}} MCF(\varPhi_{A},\varPhi_{B}) = \sum\limits_{j=1}^{M}\sum\limits_{i=1}^{N}\frac{1}{2\pi\sigma^{2}}exp\left(-\frac{\|\vec{p}_{i}-\vec{p}_{j}\|^{2}}{2\sigma^{2}}\right) \end{array} $$

where $\vec {p}_{i}\in \varPhi _{A}, i=1,2,\ldots N$ and $\vec {p}_{j}\in \varPhi _{B}, j=1,2,\ldots M$. MCF is a metric based on the conception of position sets and directly outputs the similarity of two signal distributions represented by the Dirac delta function. Note that the properties of MCF are coincident with the above basic rules. The conception of MCF is similar to cross-correlation analysis and is connected with the concept of the Parzen-Window Density Estimation [14,15]. However, MCF directly operates on the molecule positions to judge the similarity of two signal distributions, which is more conceptually straight-forward and easy to operate compared with the cross-correlation analysis of a 2D histogram.

There are several properties of MCF:

1. Reciprocity: If $\vec {p}_{i}$ contributes value to the PMCF of $\vec {p}_{j}$, $\vec {p}_{j}$ will contribute equal value to the PMCF of $\vec {p}_{i}$.
2. Additivity: Every molecule position contributes to the PMCF of a certain region. If there are several difference regions, the molecule will have contribution to each.
3. Regionally restraint: The output of the kernel function of MCF declines to zero when the value of the distance exceeds a threshold.

### Drift correction

The super-resolution image is reconstructed from a series of frames. For convenience in our later discussion, we define the positions of all the frames, i.e., the positions composing the super-resolution image, by Φ_*all*_, and define the positions of the *i*th frame by Φ_*fr*(*i*)_. Therefore, Φ_*fr*(*i*)_ is a subset of Φ_*all*_, i.e., ∀*i*,Φ _*fr*(*i*)_⊆Φ _*all*_. The position sets of several successive frames can be combined (temporally binned) into a union. If the frames are binned at an interval of *t*, we defines the union set of the *i*th interval by Φ_*T*(*i*)_, i.e., $\varPhi _{T(i)}=\varPhi _{fr(i\cdot t-t+1)}\bigcup \varPhi _{fr(i\cdot t-t+2)}\bigcup \ldots \bigcup \varPhi _{fr(i\cdot t)}$.

Assuming there are two position sets Φ_*A*_ and Φ_*B*_ ($\vec {p}_{i}\in \varPhi _{A}, i=1,2,\ldots N$ and $\vec {p}_{j}\in \varPhi _{B}, j=1,2,\ldots M$), the cost function *MCF*(*L*) of drift correction is given by:

(7) $$\begin{array}{@{}rcl@{}} MCF(L) = \sum\limits_{j=1}^{M}\sum\limits_{i=1}^{N}\frac{1}{2\pi\sigma^{2}}exp\left(-\frac{\|L(\vec{p}_{j})-\vec{p}_{i}\|^{2}}{2\sigma^{2}}\right) \end{array} $$

where *L*(·) is a transformation function, Φ_*B*_ is movable and Φ_*A*_ is fixed. Our aim is to find an *L*(·) that maximizes the MCF. The choice of the shape of *L*(·) depends on the method of image acquisition. In most applications, the drift is assumed to be linear [9,10], so we will make that assumption (though affine projection may occur in some cases). Therefore, in the following contents, the transformation is defined as linear, i.e., $L(\vec {p})=\vec {p}+\vec {d}$, where $\vec {d}=(\alpha,\beta)^{T}$, *α* is the drift compensation of the *x*-axis and *β* is the drift compensation of the *y*-axis.

**Figure 2:**
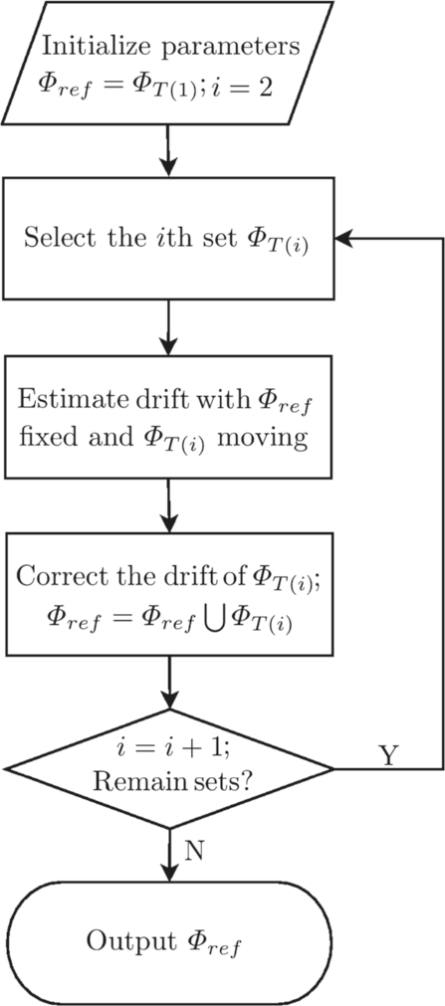
Flowchart of the incremental drift correction algorithm. **Flowchart of the incremental drift correction algorithm.**

As discussed in [10], there is a difficulty in choosing the reference image when doing drift correction based on the cross-correlation analysis of a 2D histogram. To depress the deviation of the estimation error, we propose an incremental registration algorithm based on the MCF metric. Our method is summarized in Figure 2. Firstly, the position sets of frames are binned at an interval of *t*. Our algorithm takes {Φ_*T*(*i*)_} and an empty reference set Φ_*ref*_ as input. The reference position set Φ_*ref*_ is initialized by Φ_*T*(1)_ and the counter *i* is set to two. Then position set Φ_*T*(*i*)_ is selected to compare with Φ_*ref*_ and the sample drift is estimated. The drift of Φ_*T*(*i*)_ will be compensated and the positions of Φ_*T*(*i*)_ will be merged into Φ_*ref*_. Repeat the selection and drift compensation process until all the subset are corrected. Finally, the reference position set Φ_*ref*_ will be output as the data for the super-resolution image reconstruction. Many techniques could be utilized to solve the maximum problem, for example, Grid Searching, Conjugate Gradient. We have implemented the algorithm in C++, based on GNU Scientific Library (GSL); it is freely available from the authors upon request.

In the process of drift estimation between Φ_*T*(*i*)_ and Φ_*ref*_, all the information from the previously corrected position sets are used. Therefore, according to the study of Geisler [10], the deviation of drift estimation error can be controlled by this incremental strategy of our algorithm. Two parameters, the *σ* of MCF and the temporal binning interval *t*, should be initialized. According to the Nyquist sampling theorem, the value of *σ* should be more than double the standard deviation of the molecular localization uncertainty. The choice of temporal binning interval *t* is less restricted than that in the method of cross-correction analysis. In the following experiments, we prove that our method can achieve high drift correction accuracy with very few molecule positions in each Φ_*T*(*i*)_, assuming the sampling has no bias.

## Results

### Simulated data

**Figure 3:**
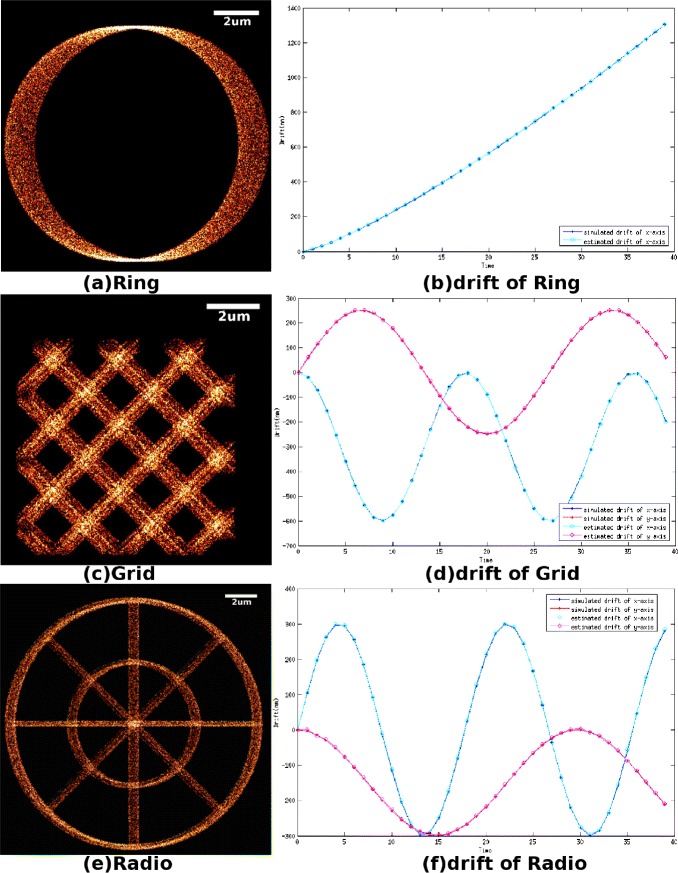
Simulated data with drift imposed on the data. **Simulated data with drift imposed on the data.** **(a)** Super-resolution image of data set Ring, which is degenerated by the drift(10 nm per pixel). **(b)** Blue solid points denote the exactly simulated values that are imposed on the Ring data set as drift; cyan cycles denote the estimated drifts that are calculated from the degenerated data set by our algorithm. **(c)** Super-resolution image of data set Grid, which is degenerated by the drift(10 nm per pixel). **(d)** Solid points denote the exactly simulated values that are imposed on the Grid data set as drift, where the blue one refers to the drift along the *x*-axis and the red one refers to the drift along the *y*-axis; cycles denote the estimated drifts that are calculated from the degenerated data set by our algorithm, where the cyan one refers to the drift along the *x*-axis and the pink one refers to the drift along the *y*-axis. **(e)** Super-resolution image of data set Radio, which is degenerated by the drift(10 nm per pixel). **(f)** Solid points denote the exactly simulated values that are imposed on the Radio data set as drift, where the blue one refers to the drift along *x*-axis and the red one refers to the drift along *y*-axis; cycles denote the estimated drifts that are calculated from the degenerated data set by our algorithm, where the cyan one refers to the drift along the *x*-axis and the pink one refers to the drift along the *y*-axis.

Three simulated data sets have been designed to evaluate the performance of our method, as showed in Figure 3. The first structure, named ‘Ring’, consists of 40,000 molecules randomly distributed on a ring structure with a width of 200 nm. The molecules are divided into *T*=40 equal step length bins (i.e., there are 40 groups of molecules and in each group there are 1000 molecules) with drift imposed along the x-axis. The linear drift in time *t*_*j*_,*j*=1,2,…*T* is illustrated in Figure 3(b) and the structure after the drift is shown in Figure 3(a). The second structure, named ‘Grid’, consists of 60,000 molecules distributed on a grid structure, the thickness of the lines of the grid is 120 nm. The molecules are divided into *T*=40 equal step length bins (i.e., there 40 groups of molecules and in each group there are 1500 molecules) with drift imposed along x-axis and y-axis. The drift of x-axis and y-axis in time *t*_*j*_,*j*=1,2,…*T* is illustrated in Figure 3(d) and the structure after the drift is shown in Figure 3(c). The third structure, named ‘Radio’, consists of 60,000 molecules labeled on a structure composed of two ring structures and several lines radiating from the center, where the thickness of the ring structure is 120 nm and the thickness of the line is 80 nm. The molecules are divided into *T*=40 equal step length bins (i.e., there are 40 groups of molecules and in each group there are 1500 molecules) with drift imposed along x-axis and y-axis. The drift of x-axis and y-axis in time *t*_*j*_,*j*=1,2,…*T* is illustrated in Figure 3(f) and the structure after drift is shown in Figure 3(e).

**Figure 4:**
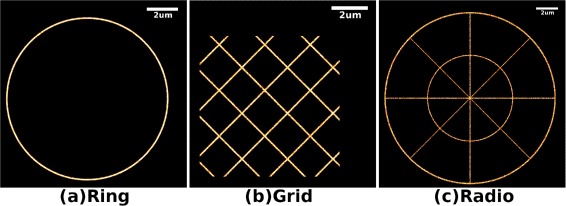
Super-resolution images of drift corrected data (10 nm per pixel). **Super-resolution images of drift corrected data (10 nm per pixel).** **(a)** Super-resolution image of data set Ring which is recovered from the data set illustrated in Figure 3(a). **(b)** Super-resolution image of data set Grid which is recovered from the data set illustrated in Figure 3(c). **(c)** Super-resolution image of data set Radio which is recovered from the data set illustrated in Figure 3(e).

Figure 4 demonstrates the molecule positions after drift correction. The estimated drift of every data set is illustrated in Figure 3. It should be noted that the drifts estimated by our algorithm almost coincide with the simulated drifts. The effectiveness of drift compensation can be evaluated by residuals, which are calculated as the difference between the drift estimated by our algorithm and the drift simulated in the experiment. The means of residual errors of Ring, Grid and Radio are 2.0 nm, 2.8 nm and 3.6 nm, respectively.

**Figure 5:**
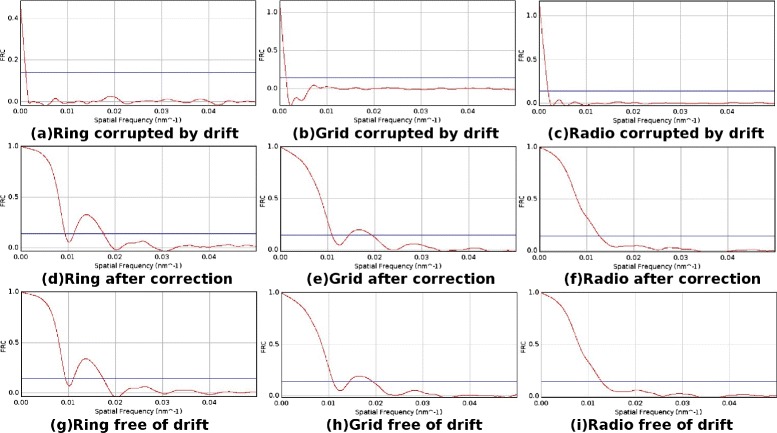
Resolution estimation by FRC. **Resolution estimation by FRC.** The red curve is FRC values and the horizontal line is the 0.143 threshold. **(a)** FRC curve of data set Ring, which is degenerated by the drift. **(b)** FRC curve of data set Grid, which is degenerated by the drift. **(c)** FRC curve of data set Radio, which is degenerated by the drift. **(d)** FRC curve of data set Ring, of which the drift is corrected by our method. **(e)** FRC curve of data set Grid, of which the drift is corrected by our method. **(f)** FRC curve of data set Radio, of which the drift is corrected by our method. **(g)** FRC curve of data set Ring, which has no drift imposed. **(h)** FRC curve of data set Grid, which has no drift imposed. **(i)** FRC curve of data set Radio, which has no drift imposed.

Quantitative analysis of the molecule positions is carried out by Fourier ring correlation (FRC) [13] for the three data sets, as illustrated in Figure 5. The resolution in super-resolution microscopy depends on the localization uncertainty, density of probe molecule positions and the sample’s spatial structure. FRC is based on the homogeneity of the distribution of position sets and could give out an estimation of resolution of the super-resolution image. Figure 5(a),(b) and (c) illustrate the FRC curves of the data sets Ring, Grid and Radio that are corrupted by sample drift. Figure 5(d),(e) and (f) illustrate the FRC curves of the data sets Ring, Grid and Radio that are corrected by our method. Figure 5(g),(h) and (i) illustrate the FRC curves of the data sets Ring, Grid and Radio that are free from drift, i.e., the original data sets that have not had drift imposed. For low spatial frequencies, the FRC curve is close to unity; for high spatial frequencies, noise dominates the data and the FRC decays to 0. The fixed threshold for determining the resolution is set to 0.143, where the value of the FRC curve announces the corresponding resolution of the super-resolution image [13]. The steeper the FRC curve is, the lower the resolution of the data. The FRC resolution of Ring corrected by our method is 106.7 nm, while the FRC resolution of Ring after drift is 890.0 nm. The FRC resolution of Grid corrected by our method is 91.4 nm, while the FRC resolution of Grid after drift is 744.0 nm. The FRC resolution of Radio corrected by our method is 78.6 nm, while the FRC resolution of Radio after drift is 524.9 nm. As a comparison, the FRC resolutions of the original data sets that are free of sample drift are 106.4 nm, 91.2 nm and 77.7 nm for Ring, Grid and Radio, respectively. It should be noted that the FRC curve of data sets restored by our method are very close to the original data sets, which proves the effectiveness of our method.

**Figure 6:**
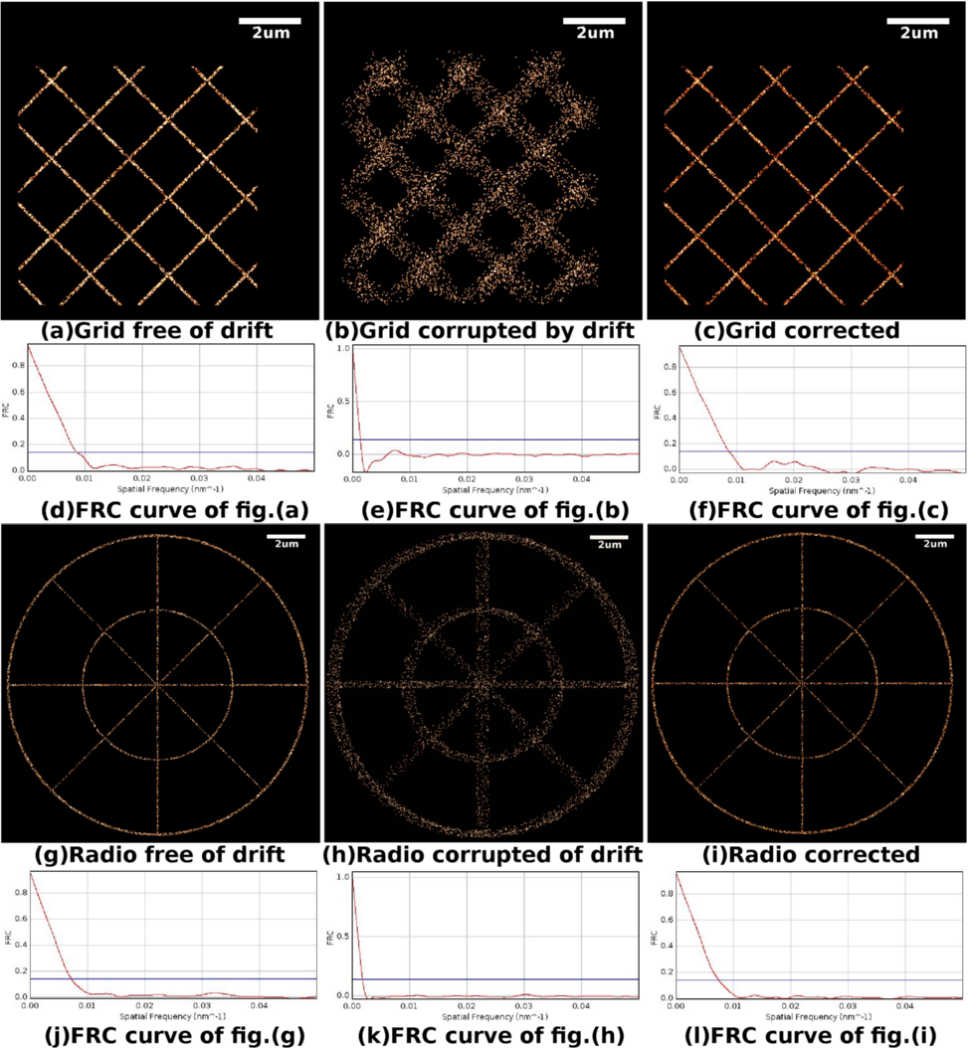
Drift correction of the data set where there are 150 molecule positions in each temporal bin. **Drift correction of the data set where there are 150 molecule positions in each temporal bin.** **(a)** Super-resolution image of data set Grid, which has no drift imposed. **(b)** Super-resolution image of data set Grid, which is corrupted by drift. **(c)** Super-resolution image of data set Grid, of which the drift is corrected by our method. **(d)** FRC curve of the data set in sub-fig(a). **(e)** FRC curve of the data set in sub-fig(b). **(f)** FRC curve of the data set in sub-fig(c). **(g)** Super-resolution image of data set Radio, which has no drift imposed. **(h)** Super-resolution image of data set Radio, which is corrupted by drift. **(i)** Super-resolution image of data set Radio, of which the drift is corrected by our method. **(j)** FRC curve of the data set in sub-fig(g). **(k)** FRC curve of the data set in sub-fig(h). **(l)** FRC curve of the data set in sub-fig(i).

Because the drift correction based on MCF does not need to bin the molecule positions into a 2D histogram, our algorithm has the potential to estimate drift with a very small number of positions in each temporal bin. Figure 6 illustrates the results of an experiment based on the data sets Grid and Radio, where all the conditions are the same as the previous experiment except that there are in total 6,000 molecule positions in every data set (150 positions for each bin). Figure 6(a) (g) illustrate the super-resolution images of Grid and Radio, respectively, which are free from drift. Figure 6(b) (h) illustrate the super-resolution images of the data sets that are corrupted by drift. 6(c) (i) illustrate the super-resolution images of the data sets that are corrected by our method. Figure 6(d) (e) (f) (j) (k) (l) are the corresponding FRC curves. It should be noted that the super-resolution images of the corrected data sets is very close to that of sets free of drift, and their FRC curves are very similar. Here, the FRC resolution of Grid corrected by our method is 115.7 nm, while the FRC resolution of Grid after drift is 717.9 nm. The FRC resolution of Radio corrected by our method is 134.8 nm, while the FRC resolution of Radio after drift is 553.2 nm. As a comparison, the FRC resolutions of the data sets that are free of sample drift are 115.7 nm and 133.4 nm for Grid and Radio, respectively. The resolution of the data corrected by our method is almost the same as the drift free ones. To further explain the range of possible applications, an experiment performed in a critical situation (in total 1,600 molecule positions in every data set, i.e., 40 positions for each bin) has been illustrated in the Appendix.

**Figure 7:**
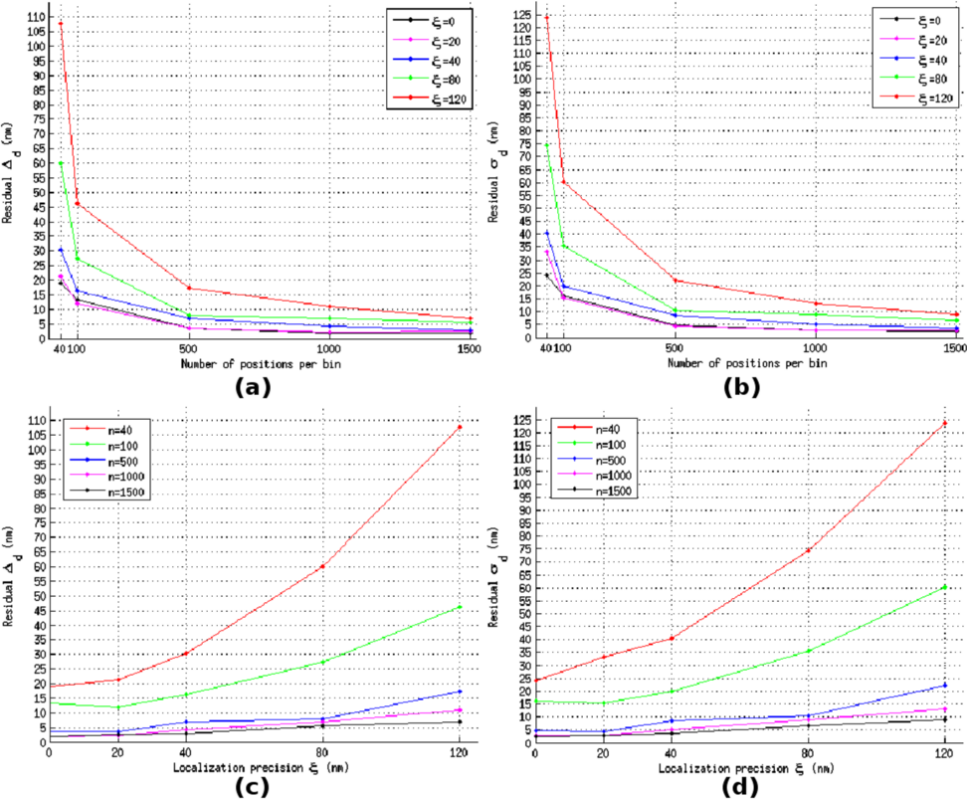
The values of the mean *Δ*_*d*_ and the standard deviation *σ*_*d*_ of residual over the *T*=40 time intervals for the data set Grid, as a function of molecule number per temporal bin *n* and localization precision *ξ*. **The values of the mean** ***Δ***_***d***_ **and the standard deviation** ***σ***_***d***_ **of residual over the** ***T*** **=40 time intervals for the data set Grid, as a function of molecule number per temporal bin** ***n*** **and localization precision** ***ξ*** **.** **(a)** Curve of residual *Δ*_*d*_ in different localization precisions *ξ*, as a function of molecule numbers *n*. **(b)** Curve of residual’s standard deviation *σ*_*d*_ in different localization precisions *ξ*, as a function of molecule numbers *n*. **(c)** Curve of residual *Δ*_*d*_ in different molecule numbers *n*, as a function of localization precisions *ξ*. **(d)** Curve of residual’s standard deviation *σ*_*d*_ in different molecule numbers *n*, as a function of localization precisions *ξ*.

**Figure 8:**
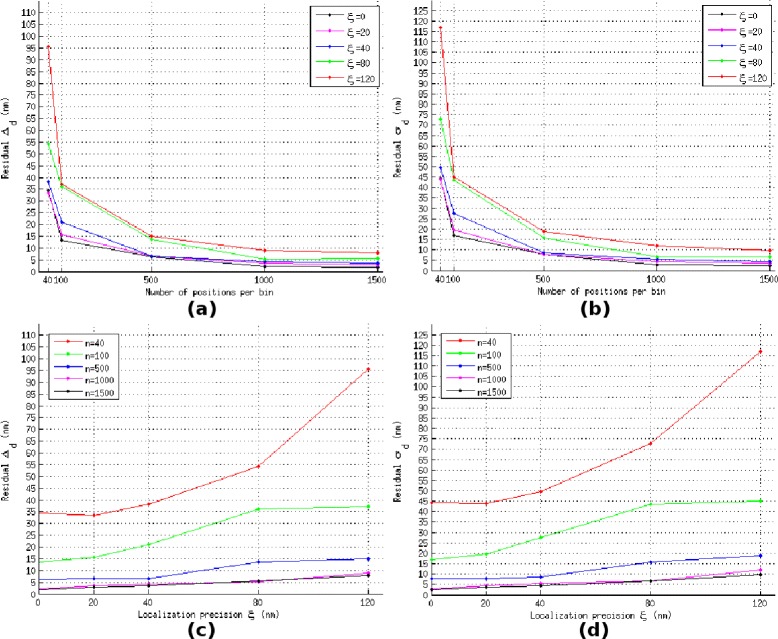
The values of the mean *Δ*_*d*_ and the standard deviation *σ*_*d*_ of residual over the *T*=40 time intervals for the data set Radio, as a function of molecule number per temporal bin *n* and localization precision *ξ*. **The values of the mean** ***Δ***_***d***_ **and the standard deviation** ***σ***_***d***_ **of residual over the** ***T*** **=40 time intervals for the data set Radio, as a function of molecule number per temporal bin** ***n*** **and localization precision** ***ξ*** **.** **(a)** Curve of residual *Δ*_*d*_ in different localization precisions *ξ*, as a function of molecule numbers *n*. **(b)** Curve of residual’s standard deviation *σ*_*d*_ in different localization precisions *ξ*, as a function of molecule numbers *n*. **(c)** Curve of residual *Δ*_*d*_ in different molecule numbers *n*, as a function of localization precisions *ξ*. **(d)** Curve of residual’s standard deviation *σ*_*d*_ in different molecule numbers *n*, as a function of localization precisions *ξ*.

It is also known that the position localization precision and molecule numbers per temporal bin affect the performance of drift compensation [9]. Experiments in different molecule numbers per temporal bin (denoted by *n*) and localization precision (denoted by *ξ*) of data set Grid and Radio were carried out to illustrate the impact. The mean *Δ*_*d*_ and the standard deviation *σ*_*d*_ of residual over the *T*=40 temporal intervals were calculated for each simulation. Figures 7 and 8 illustrate the curves of *Δ*_*d*_ and *σ*_*d*_ as a function of *n* and *ξ*. For both Figures 7 and 8, sub-fig (a) (c) represent the same *Δ*_*d*_ and sub-fig (b) (d) represent the same *σ*_*d*_, in two different views. The tendencies of the mean residual and the standard deviation of the residual under changes in *n* and *ξ* are consistent. Judging from Figure 7(a) and (b), we can find that our method behaves well in the situations in which the localization precision *ξ*≤40 for data set Grid. Under the condition that localization precision *ξ*≤80 and molecule numbers per bin *n*≥500, the accuracy of drift compensation by our method can reach to 2∼10 nm. Comparing Figure 8(a) (b) to Figure 7(a) (b), we find that the performance of our method in data set Radio is not as good as that in Grid when *ξ*=80. The reason why there is such a difference is that when the localization precision reaches *ξ*=80, the localization uncertainty is so large compared to the width of the line structure in Radio (80 nm) that it degenerates the sampling of the molecule distribution. Our method performs not good when *ξ*=120 nm (the width of line in Grid) and the molecule number per bin *n* is very small. The performance of our method is impacted by bias sampling or mal-sampling, because our method is based on the direct recovery of position distributions. Nevertheless, according to recent studies [1,13], localization precisions are usually controlled to 10∼35 nm in 2D, which makes the low localization precision not a problem for our method. Judging from sub-fig (c) and (d), we can find that *n*=500 is sufficient to support a good performance of our method. Additionally, if the localization precision *ξ*≤20 nm, judging from Figures 7 and 8, we can conclude that our method can compensate the sample drift to a residual of 20∼40 nm with 40 molecule positions per temporal bin and to a residual of about 15 nm with 100 molecule positions per temporal bin. Readers who are interest in the details of the performance of our method in the cases where *n*=40 and *ξ*=0 nm can refer to the Appendix.

### Super-resolution experimental data

To confirm the effectiveness of our drift correction method for practical applications, real super-resolution experimental data is utilized.

COS-7 cells were cultured in DMEM complete medium (Gibco) supplemented with 10*%* fetal bovine serum and maintained at 37°C and 5*%* CO2 in a humidified incubator (Thermo). The cells were fixed with 3*%* (w/v) paraformaldehyde and 0.5*%* glutaraldehyde in PBS for 15 to 40 min at 37°C and washed 3 to 5 times with filtered PBS. After that, the fixed COS-7 cells were permeabilized for 10 min with 0.1*%* Triton X-100 and then blocked in 5*%* bovine serum albumin (BSA, AMRESCO) diluted in PBS for 60 min. The mouse anti- *β* tubule monoclonal antibody (Sungenebiotech) was diluted 1:200 in PBS containing 2.5*%* BSA and added to the COS-7 cells in 37°C incubator for 60 min. After three times rinsing with PBS, COS-7 cells were then incubated for another 60 min with the Alexa Fluor 647 labeled rabbit anti-mouse secondary antibody (Invitrogen), which was also diluted 1:200 in PBS. At last, the cells were rinsed four times with PBS and kept in a dark place. The STORM imaging buffer contained imaging buffer base (10*%* glucose (m/v), 50 mM Tris (pH 8.0) and 10 mM NaCl), an oxygen scavenger system (0.5 mg ml ^−1^ glucose oxidase (Sigma-Aldrich), 40 *μ*g ml ^−1^ catalase (Sigma-Aldrich)) and 10 mM MEA. Two fluorescent beads were embedded on the surface of the sample.

STORM imaging of microtubules was performed as described in [16]. We used an Olympus IX71 inverted microscope equipped with a 150×1.45 numerical aperture (NA) oil objective (Olympus PLAN APO). Two lasers (405 nm and 647 nm (OBIS, Coherent)) were controlled by an acousto-optic tunable filter (AA Optoelectronic). For excitation, the power of the 647 nm laser was 29.71 mW, measured near the rear pupil of the objective. The intensity of the 405 nm laser, typically 10-30 *μ*W, was adjusted so that a low density of molecules was activated at each frame. A *λ*/4 plate was used to produce circular polarization excitation light. The fluorescence signals were acquired using an electron-multiplying charge-coupled device (EMCCD) camera (Andor iXon DU-897). The images were acquired at a frame rate of 50 Hz and the EM gain of EMCCD was set to 300. Fluorescent beads were embedded into the sample for our convenience in comparing them. The super-resolution image reconstruction was performed as described in [17]. In order to minimize the influence of the background, TIRF (total internal reflection) illumination was used in this study.

**Figure 9:**
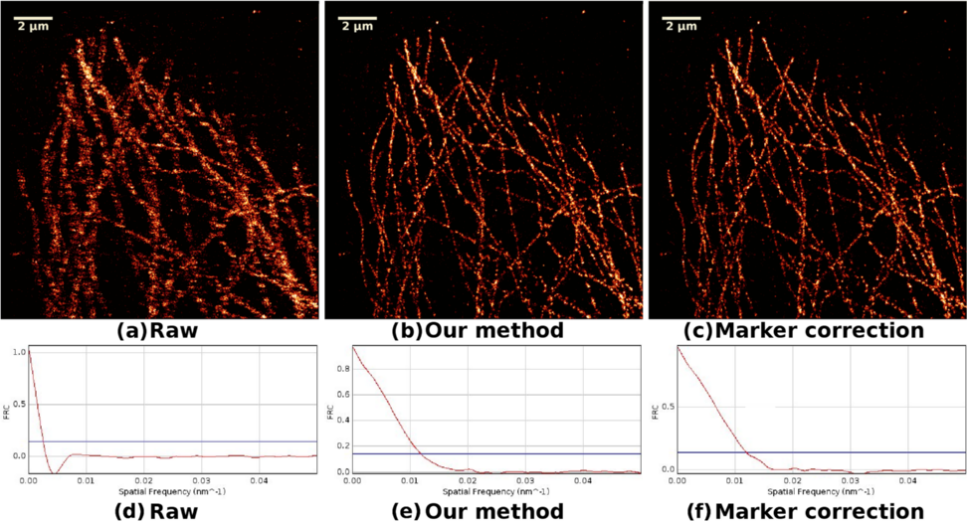
Experimental data. **Experimental data.** **(a)** Super-resolution image of experimental data set, of which the drift has not been corrected. **(b)** Super-resolution image of experimental data set, of which the drift is corrected by our method. **(c)** Super-resolution image of experimental data set, of which the drift is corrected by method based on beads. **(d)** FRC curve of the data set in sub-fig(a). **(e)** FRC curve of the data set in sub-fig(b). **(f)** FRC curve of the data set in sub-fig(c).

5000 frames were recorded during the experiment and in total 57,702 molecular positions were extracted. Drift correction based on both beads and our method was carried out. Every 500 frames were binned into a “big” time interval. The bead positions were determined in each frame within the 500 frame bin interval and their cumulative localizations were used as a reference marker in drift correction. The experimental result is illustrated in Figure 9. The raw image without drift correction is illustrated in Figure 9(a). The structure corrected by our method is illustrated in Figure 9(b) and the structure corrected by fluorescent beads is illustrated in Figure 9(c). The FRC curves for the three structures are also illustrated in Figure 9(d), (e) and (f). The FRC resolution of the raw data is 393.4 nm, the resolution of the data corrected by our method is 84.3 nm and the resolution of the data corrected by marker is 83.9 nm. We find that the result of our method is very close to that of the fluorescent beads. In addition, the recent work [10] has confirmed that for some special scenarios, using the biological data itself could achieve better results than the use of beads, which may move or fade during data acquisition. Thus, generally, our method could be more feasible than the method using fluorescent beads.

## Discussion and conclusion

In this paper, we proposed a new metric based on distance minimums to cope with the drift correction in single-molecule imaging. Our method is based on the restoration of the distribution of molecular position. There are two advantages of our method. First, because our method does not require space binning of positions of molecules but directly operates on the positions, it is more natural to single molecule imaging techniques. Second, our method estimates the drift with a small number of positions in each temporal bin, which may extend the potential application of our metric. The result of simulated test data proved the effectiveness of our method. Our method can be easily extended into 3D super-resolution photography, and the Gaussian kernel can also be replaced in some special situations.

## Appendix

**Figure 10:**
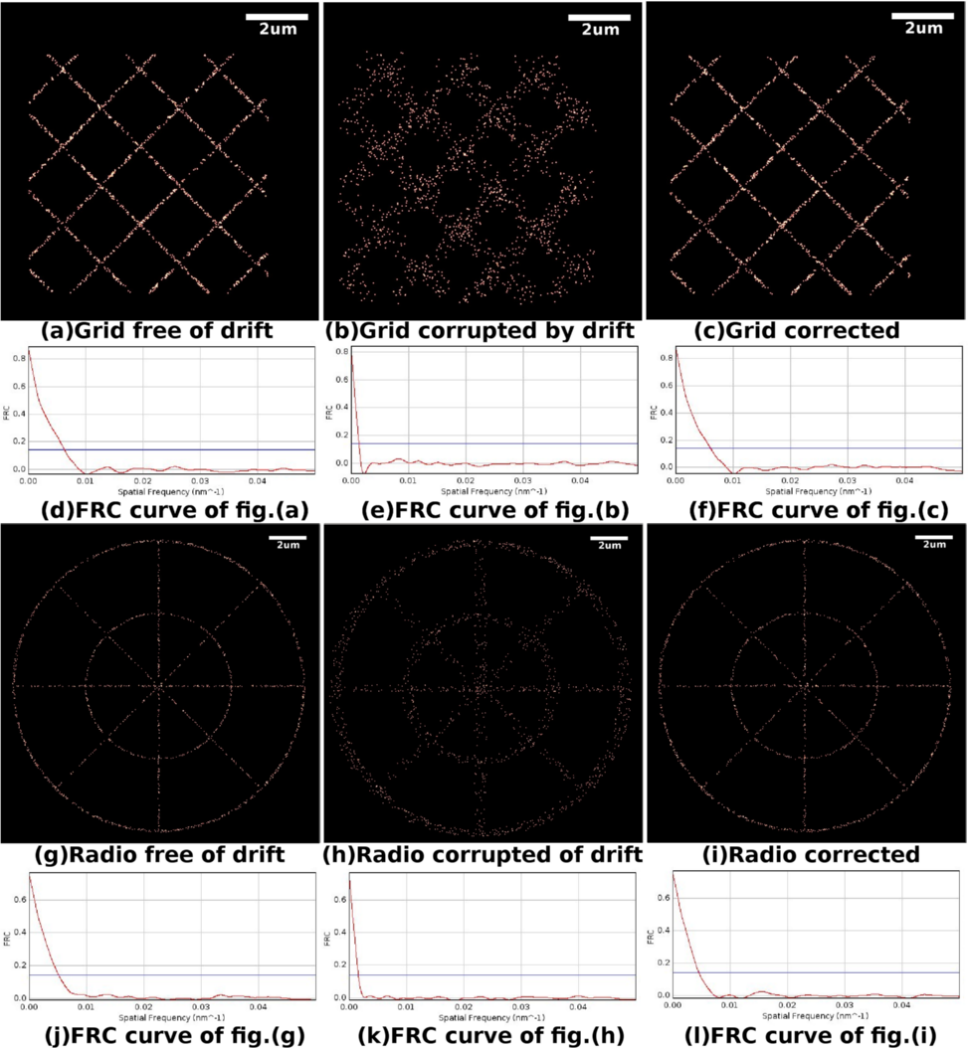
Drift correction of the data set where there are 30 molecule positions in each temporal bin. **Drift correction of the data set where there are 30 molecule positions in each temporal bin.** **(a)** Super-resolution image of data set Grid, which has no drift imposed. **(b)** Super-resolution image of data set Grid, which is corrupted by drift. **(c)** Super-resolution image of data set Grid, of which the drift is corrected by our method. **(d)** FRC curve of the data set in sub-fig(a). **(e)** FRC curve of the data set in sub-fig(b). **(f)** FRC curve of the data set in sub-fig(c). **(g)** Super-resolution image of data set Radio, which has no drift imposed. **(h)** Super-resolution image of data set Radio, which is corrupted by drift. **(i)** Super-resolution image of data set Radio, of which the drift is corrected by our method. **(j)** FRC curve of the data set in sub-fig(g). **(k)** FRC curve of the data set in sub-fig(h). **(l)** FRC curve of the data set in sub-fig(i).

Figure 10 illustrates the results of an experiment based on data sets Grid and Radio, where all the conditions are the same as the previous experiment except that there are in total 1,600 molecule positions in every data set (40 positions for each bin). Here, the FRC resolution of Grid corrected by our method is 164.5 nm, while the FRC resolution of Grid after drift is 730.9 nm. The FRC resolution of Radio corrected by our method is 214.4 nm, while the FRC resolution of Radio after drift is 620.3 nm. As a comparison, the FRC resolutions of the data sets that are free of sample drift are 158.1 nm and 193.2 nm for Grid and Radio respectively. Our method performed well for data set Net, but barely satisfactorily in data set Grid. The reason why there is such a difference in behavior of our method on the two sets is that the sampling is too sparse and has introduced bias in the data set Radio. Though our method has not corrected all the sample drift under this condition, our method has corrected the majority of the drift in the situations where the cross-correlation method barely works. This experiment demonstrates that our method can correct sample drift in a very critical situation. Nevertheless, 40 positions per temporal bin may not happen in a real experiment and the experiment in this Appendix is only intended to demonstrate the range of potential applications of our method.
